# Massive heterotopic ossification associated with late deficits in posterior wall of acetabulum after failed acetabular fracture operation

**DOI:** 10.1186/1471-2474-14-368

**Published:** 2013-12-26

**Authors:** Yuntong Zhang, Yang Xie, Shuogui Xu, Chuncai Zhang

**Affiliations:** 1 Department of orthopaedics, Changhai Hospital, Second Military Medical University, Shanghai 200433, China

**Keywords:** Heterotopic ossification, Late bone defects, Posterior wall, Acetabulum, Acetabular fracture

## Abstract

**Background:**

Heterotopic ossification is a common postoperative complication of acetabular fracture. However, functionally significant heterotopic ossification with associated late bone defects in the posterior wall of the acetabulum is rare and challenging to treat. When heterotopic ossification is a late complication of failed acetabular fracture operation, it is disabling and may only be treated by THA. THA is highly susceptible to premature failure in young and active patients and may require numerous revisions.

**Case presentation:**

This article describes a 40-year-old man with massive heterotopic ossification associated with late bone defects in the posterior wall of the acetabulum after a failed acetabular fracture operation. The primary fracture type was a 62-A2.3 fracture according to the AO/OTA Classification.Surgical excision and anatomical reconstruction of the acetabular wall using heterotopic ossific bone were performed 10 months after the fracture repair. Postoperatively, indomethacin was administered for prophylaxis against recurrence of heterotopic ossification, and hip range of motion was progressively increased. At 5 years and 6 months follow-up, the patient’s pain was relieved and hip function had recovered. Though radiography and CT showed minimal subchondral cysts and mild joint-space narrowing, there was no evidence of graft resorption, progressive posttraumatic osteoarthritis or necrosis of the femoral head.

**Conclusion:**

To the authors’ knowledge, this is the first case of such a challenging condition. Although it is an extremely rare case, it provides an attractive option for avoiding THA, as the long-term follow-up shows a satisfactory outcome.

## Background

Heterotopic ossification (HO), the development of bone outside its normal location in the skeleton, is a common postoperative complication of acetabular fractures [1]. Irradiation and indomethacin have been shown to be effective in the prevention of severe heterotopic ossification [2]. However, once formed, heterotopic bone can be managed only with surgical excision [3]. In one study, functionally significant heterotopic ossification (Brooker et al. class III or IV) developed in 23% of those patients who did not receive regular prophylaxis [1]. Although surgical excision of heterotopic ossification has been reported with satisfactory results [4,5], the management of disabling HO with associated bone defects in the posterior wall of the acetabulum is a challenge for surgeons and has not been reported to the best of our knowledge.

We describe a rare case of massive HO surrounding the hip joint with associated bone defects in the posterior wall of the acetabulum following a failed operation of acetabular fractures. The management and outcome, five years and six months after the excision, as well as measures to prevent recurrence are discussed. Informed consent for participation in the study was obtained from the patient.

## Case presentation

**Figure 1:**
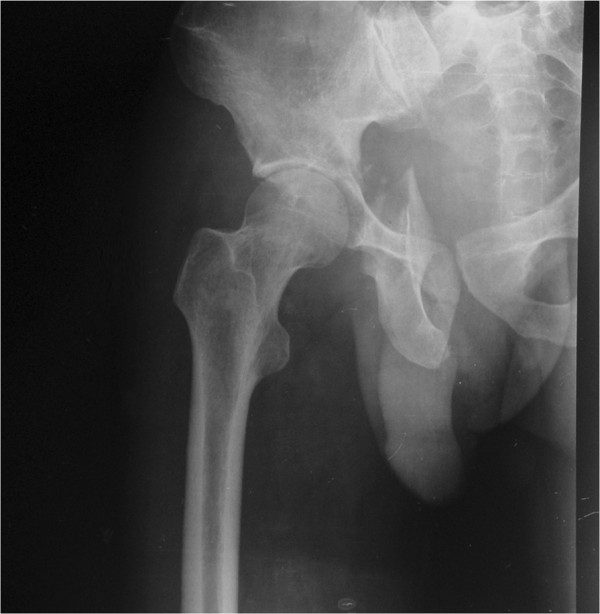
An AP view of the right hip after injury shows a posterior wall and column fracture of the right acetabulum and a fracture of the ipsilateral inferior ramus of pubis.

**Figure 2:**
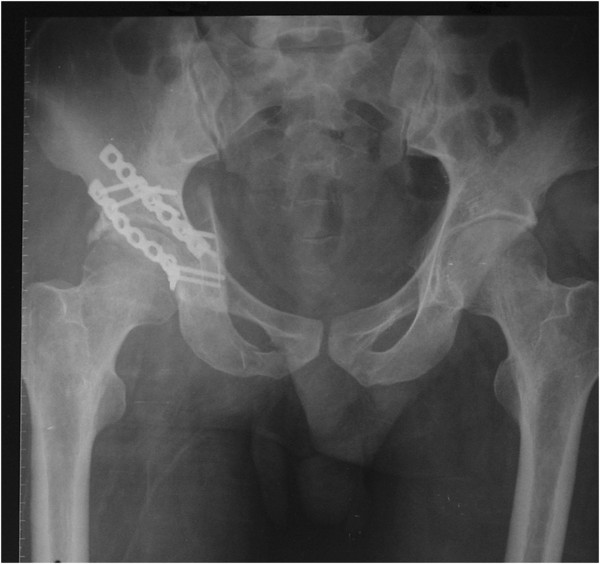
An AP view of the pelvis after initial operation shows that two lock reconstruction plates were planted, but the displaced posterior column fracture was not restored anatomically and the comminuted posterior wall fracture was not fixed rigidly.

**Figure 3:**
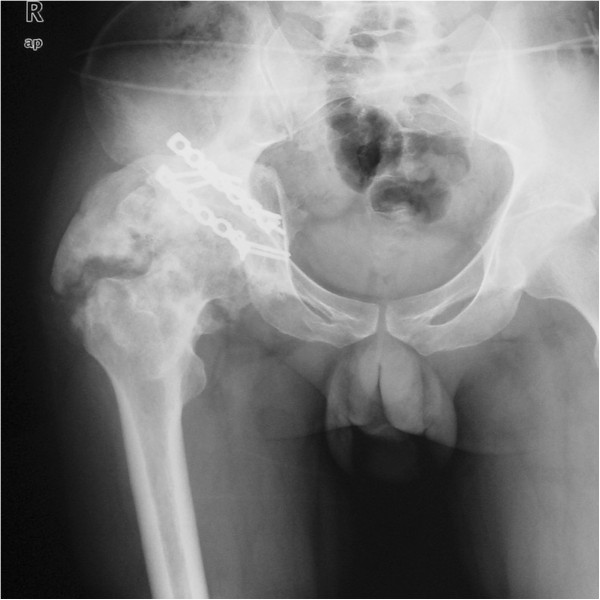
An AP view of the pelvis after 10 months shows a massive osseous lesion in the posterolateral aspect of the acetabulum.

**Figure 4:**
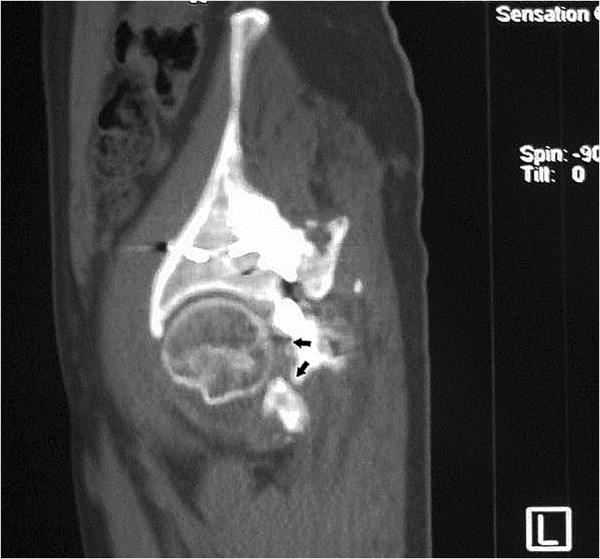
**Coronal CT scan shows incongruent reduction of the hip joint and mal-union in the posterior wall and column of the acetabulum (arrows).** However, more than 50% of joint space was maintained and no evidence of collapse or necrosis in femoral head was revealed.

**Figure 5:**
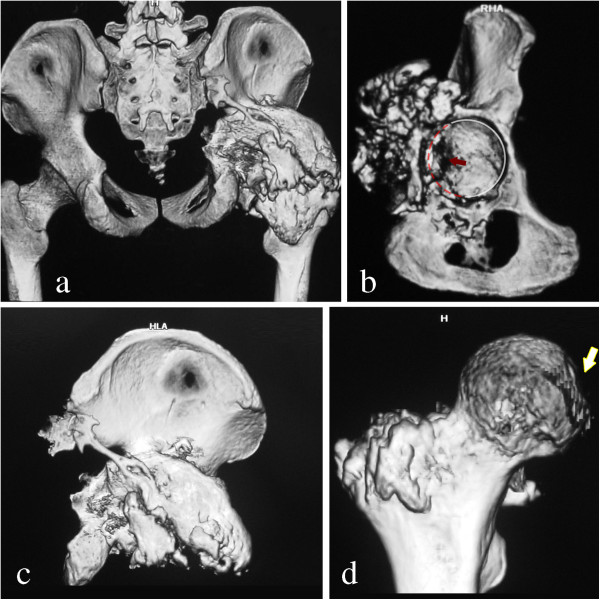
**Three-dimensional CT reconstruction. (a)** The massive osseous lesion in the posterolateral aspect of the acetabulum, partially connected with the posterior column, and surrounding the whole posterior hip joint from the roof of the acetabulum to the intertrochanteric line. **(b,c)** Bone defects and joint incongruence were revealed in the posterior wall (red dotted line and arrow in **b**). **(d)** Old impaction and degeneration exist at the posteroinferior portion of femoral head (white arrow in **d)**. However, the weight-bearing portion of the femoral head had a normal appearance.

A forty-year-old man presented with a posterior column fracture of the acetabulum and ipsilateral inferior ramus of the pubis fracture associated with enterorrhexis of the rectum due to a traffic accident on Mar. 25^th^, 2006. Emergency repair of the rectum was performed immediately after the injury. During emergency exploration and repair, the pelvic fracture was determined to have no relation to the rectal injury, thus defining the fracture as a closed fracture. The primary acetabular fracture was a 62-A2.3 fracture according to the AO/OTA Classification. After eleven days in stabilized condition, the patient accepted an open reduction and internal fixation surgery through a posterolateral approach in a local hospital. However, the fracture was not reduced after the first surgery. The postoperative radiographic images (Figure 1) show that two lock reconstruction plates were planted, but the displaced posterior column fracture was not restored anatomically, and the comminuted posterior wall fracture was not fixed rigidly. On Jan.10^th^, 2007, the patient presented to our outpatient clinic with increasing hip pain with weight-bearing and severe claudication. On physical examination, the affected limb was 3 cm shortened. Range of motion of the hip was limited to 20° in flexion, 0° in extension, 15° in adduction, and 0° in abduction. The radiographic images (Figures 2, 3, 4 and 5), including CT scans and three-dimensional reconstruction, revealed posterior subluxation and mal-union of the posterior column fracture, nonunion and bone defects in the posterior wall, and old impaction and degeneration at the posteroinferior portion of femoral head. Moreover, a massive osseous lesion existed in the posterolateral aspect of acetabulum, partially connected to the posterior column and surrounding the whole posterosuperior hip joint from the roof of the acetabulum to the intertrochanteric line. The osteoarthritic changes, including joint space narrowing, osteophyte formation, and subchondral lesions, were observed in the posterior area of the hip joint. However, more than 50% of the joint space remained, and the CT scan and three-dimensional reconstruction showed no evidence of collapse or necrosis. According to the medical record, no related therapy, including pharmacological or localized irradiation, was used for the prevention of heterotopic ossification.

**Figure 6:**
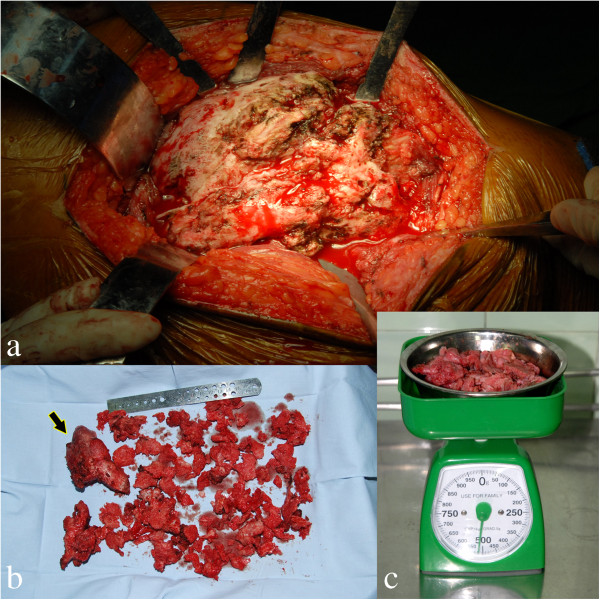
**Intra-operative photograph. (a)** The large osseous lesionadhering to the surrounding tissue and **(b)** bone was removed en bloc after meticulous release. **(c)** The total weight of heterotopic ossific bone excised was about 515g.

**Figure 7:**
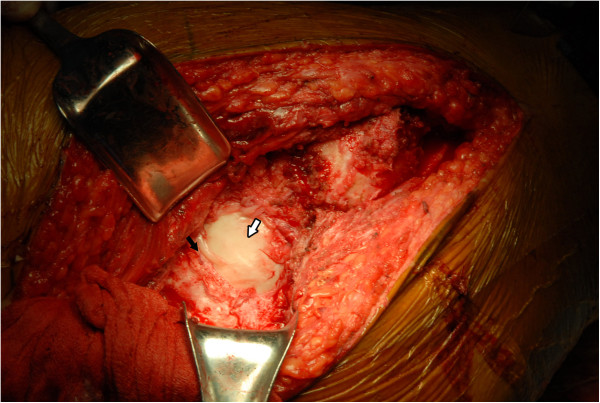
**An intraoperative picture shows a large defect (black arrow) in the posterior wall of the acetabulum.** The white arrow indicates the femoral head.

**Figure 8:**
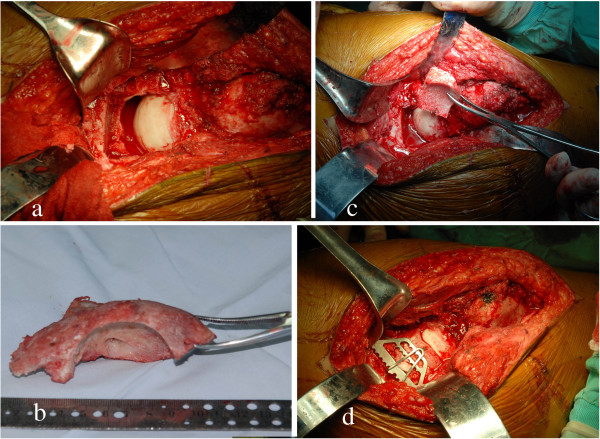
**Intra-operative photograph. (a,b)** The posterior acetabular wall defect was reconstructed with structured autograft harvested from the largest heterotopic ossific bone using an appropriate-sized acetabular reamer. **(c)** Then, the graft was placed in the deficiency. **(d)** Acetabular tridimensional memory alloy fixation system was used for internal fixation.

To establish the diagnosis and relieve symptoms, the patient underwent operative excision of the osseous lesion in the hip and anatomical reconstruction of the posterior acetabular wall using structured iliac crest autograft with a Kocher-Langenbeck approach 9 months after the primary injury. The greater trochanter osteotomy was used to allow better access to the superior acetabulum. The large osseous lesion (Figure 6a) adhering to the surrounding tissue and bone was removed en bloc after meticulous release (Figure 6b). The total weight of heterotopic ossific bone excised was approximately 515 g (Figure 6c). The screws and plates from the former operation were removed. The sciatic nerve was identified and carefully preserved during the approach and release. A large defect was noticed after removal of the bony mass (Figure 7), and the joints were unstable on examination. The posterior acetabular wall defect was reconstructed with structured autograft (Figure 8) harvested from the largest heterotopic ossific bone using an appropriate-sized acetabular reamer. The size of acetabular reamer was determined by the diameter of the femoral head and the depth of the acetabular fossa measured by radiography. The graft was then placed in the acetabular wall defect and fixed temporarily with two K-wires. A new method called acetabular tridimensional memory alloy fixation system (ATMFS), which has been satisfactorily used for treatment of posterior wall fractures of the acetabulum, was used for internal fixation [6-8]. The procedure of planting ATMFS was exactly the same as described in the related literature [6,7]. The two K-wires were then removed, and the wound was washed and closed around a drainage tube.

The drainage tube was removed 2 days after surgery. In the postoperative period, prophylaxis for recurrence of heterotopic ossification (indomethacin 25 mg three times daily) was administered for 6 weeks. Isometric contraction training of the lower limbs was encouraged starting one day after surgery. One week after surgery, the patient was asked to initiate and gradually increase the degree of extension and flexion of the hip while supine. Partial, toe-touch weight bearing with crutches or a walker was allowed four weeks postoperatively. Complete weight bearing on the affected limb was restricted until radiography demonstrated signs of union.

**Figure 9:**
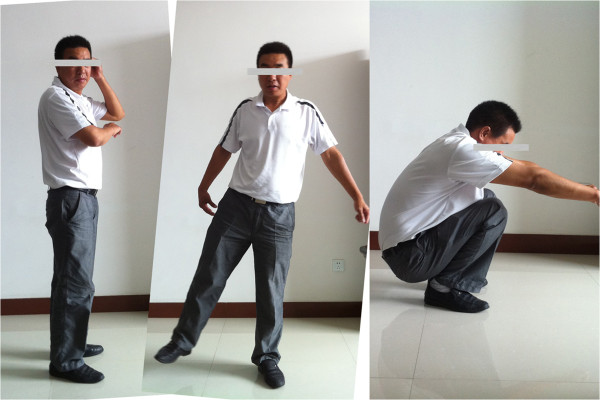
At the final follow-up examination 5 years and 6 months after the reconstructive surgery, the patient could walk by himself and the function of the injured joint was similar to the normal side.

**Figure 10:**
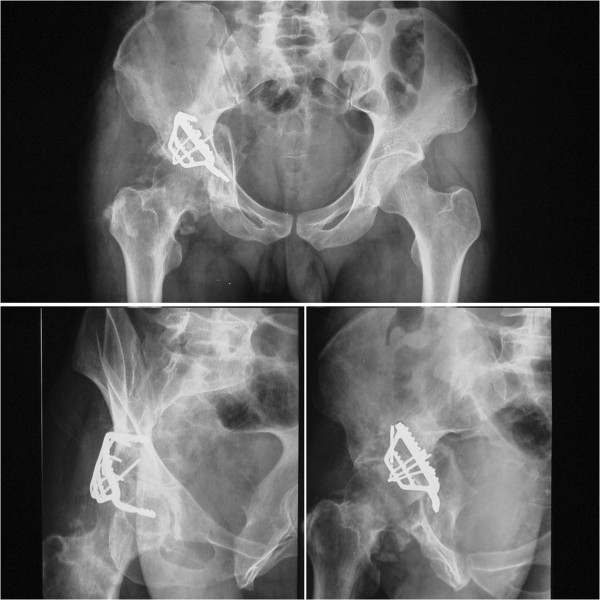
AP view and Judet oblique views obtained 5 years and 6 months postoperatively show slight recurrence of the ossified mass.

**Figure 11:**
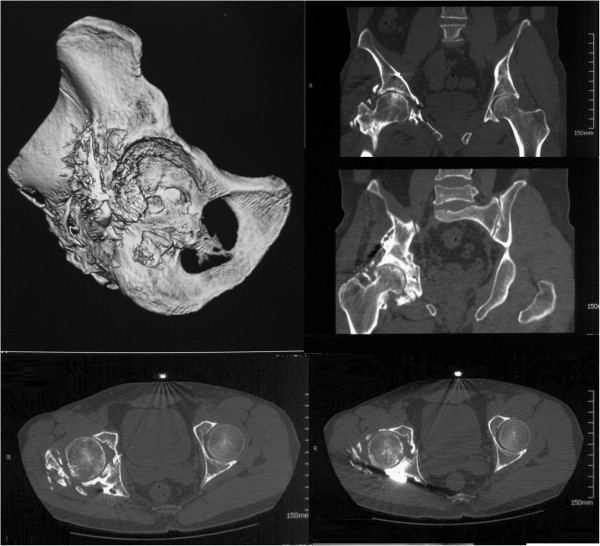
**CT scans and reconstruction at the final follow-up show more than 50% of joint space existed and the femoral head was mostly congruent with the acetabulum.** Although subchondral cysts and minimal signs of arthritis can be observed in the femoral head and acetabulum, no evidence of collapse or necrosis was found either in the femoral or acetabular subchondral bone.

At the final follow-up examination 5 years and 6 months after the reconstructive surgery, the pain was relieved, the patient could walk by himself, the function of the injured joint was similar to the normal side (Figure 9), and the range of motion of the hip was improved, with flexion improved to 120°, extension to 5°, adduction to 30° and abduction to 15°. Radiographs of the hip (Figures 10 and 11) showed slight recurrence of the ossified mass (Brooker et al. class II, mature), but with a certain distance to the joint. In addition, more than 50% of the joint space was maintained, and the femoral head was mostly congruent with the acetabulum. Although subchondral cysts and minimal signs of arthritis could be observed in the femoral head and acetabulum, no evidence of collapse or necrosis was found either in femoral or acetabular subchondral bone. The modified Merle d’Aubigne and Postel’s clinical outcome evaluation was scored as excellent. The Matta’s radiographic evaluation was scored as good.

The authors clarify that the written informed consent for participation and publication of clinical images was obtained from the patient in our study.

## Conclusion

The etiopathogenesis of HO, though incompletely understood, involves genetic abnormalities, neurologic injury, and musculoskeletal trauma [9]. The high incidence of radiographic HO and potential morbidity after acetabular surgery has led to the standardization of prophylactic therapies [10-13]. Irradiation and indomethacin are thought to be effective in the prevention of heterotopic ossification. According to recent studies, both indomethacin and radiation therapy variably decrease the rates of severe HO after acetabular surgery by 4% to 15% [14,15]. However, other studies have not verified this reduction. Sean M. et al. [16] found no reduction in HO rates after acetabular surgery with indomethacin compared with placebo.

The patient in our study did not accept any prophylaxis after the first surgery, which we conclude was the main cause of such massive heterotopic ossification. Therefore, indomethacin (25 mg three times daily) was administered for 6 weeks in the postoperative period as prophylaxis for recurrence of heterotopic ossification. Radiation therapy was not applied due to poor compliance, poor tolerance, and radiation-associated morbidities. In cases of mature hyperostotic macrodactyly, operative resection of the deposits or the osteophytes might be indicated when pain increases or the range of motion is limited.

A literature review revealed reports of satisfactory results from surgical resection of HO followed by indomethacin therapy after failed open reduction and internal fixation or total hip arthroplasty. Wick et al. [4] retrospectively analyzed the clinical effect of surgical excision of heterotopic bone after hip surgery in 21 patients. Of these patients, 19 (90.4%) had excellent relief of pain and improved hip range of motion. Only one patient (4.8%) suffered a recurrence of heterotopic bone formation. Cobb et al. [5] evaluated the outcomes of excision of heterotopic ossification after total hip arthroplasty. In all 53 cases, joint function was significantly improved. However, disabling HO with associated bone defects, mal-union in the posterior wall and incongruence of the hip joint following a failed operation of acetabular fractures have not been previously reported. Bone defect in the posterior wall of the acetabulum and joint incongruence can significantly affect the stability of the hip and lead to high incidence of posttraumatic arthritis [17]. Thus, merely the resection of the HO can hardly contribute to a favorable outcome. The usual treatment method described in the literature includes two options: one therapeutic alternative is THA [18,19]. However, posterior acetabular wall fractures occur predominantly in individuals younger than 40 years old. These exceptionally active patients are highly susceptible to premature failure of arthroplasty and may require numerous revisions throughout their lives. Another option is reconstruction of the posterior wall with the use of a graft. Among the various graft materials, the iliac crest autograft is the most common and reliable measure [20]. Nevertheless, only a few reports described reconstruction of posterior wall deficits of the acetabulum using iliac crest autograft. Daum et al. [21] first described the method in two cases of acute comminuted posterior wall acetabular fractures in 1993. The long-term functional outcome was satisfactory in one case, whereas the other case needed total hip arthroplasty after two years. Sen et al. [20] reported a series of eight cases of similar fractures where the comminuted fragments were excised and the defect in the posterior acetabular wall was reconstructed with iliac crest strut graft. The medium-term clinical outcomes were satisfactory. To our knowledge, Zha et al. [22] uniquely performed the procedure for the treatment of late posterior acetabular wall deficits following unsuccessfully managed posterior wall fractures and recommended this procedure as a noteworthy technique, especially for pediatric patients or adults without posttraumatic osteoarthritis. Compared with their reports, our technique is unique because the autograft was structured by a reamer, which has exactly the same cambered surface as the posterior acetabular surface. Furthermore, the autograft was harvested from a large heterotopic ossific bone. In addition, a Ni-Ti shaped-memory alloy device named ATMFS was used for fixation instead of screws and plates. The fixation system, as a functional metal material, has been successfully used in acetabular fractures for many years [6].

At the final follow-up, though the radiography and CT showed minimal subchondral cysts and mild joint-space narrowing, there was no evidence of graft resorption, progressive posttraumatic osteoarthritis or necrosis of the femoral head. The patient’s hip function had recovered well. We believe that the reconstruction in the presence of a concentrically reduced hip contributed to the favorable outcome. It is possible that with extension of the follow-up period, posttraumatic osteoarthritis of the hip would develop and progress, ultimately requiring THA. However, the surgical reconstruction significantly delayed the eventual THA, and the sufficient bone stock for seating of the prosthetic socket can be provided by the grafting procedure.

We report the first case of massive heterotopic ossification with associated posterior acetabular wall deficits. We also describe an audacious and unique treatment for anatomical reconstruction using heterotopic ossific bone. Although it is an extremely rare case, the long-term follow-up shows a satisfactory outcome, and it provides an attractive option for avoiding THA.

## Consent

Written informed consent was obtained from the patient for publication of this Case report and any accompanying images. A copy of the written consent is available for review by the Editor of this journal.

## Competing interest

The authors declare that they have no competing interests.

## Authors’ contributions

YZ and CZ contributed to the study concepts, literature research and manuscript preparation. CZ is the guarantor of integrity of the entire study and participated in the operation and manuscript review. YX and SX participated in literature research, data acquisition and data analysis. All authors read and approved the final manuscript.

## Pre-publication history

The pre-publication history for this paper can be accessed here:

http://www.biomedcentral.com/1471-2474/14/368/prepub
